# Screening for polystyrene nanoparticle toxicity on kidneys of adult male albino rats using histopathological, biochemical, and molecular examination results

**DOI:** 10.1007/s00441-022-03581-5

**Published:** 2022-01-28

**Authors:** Yasmine H. Ahmed, Mehrez E. El-Naggar, Maha M. Rashad, Ahmed M.Youssef, Mona K. Galal, Dina W. Bashir

**Affiliations:** 1 Cytology and Histology Department, Faculty of Veterinary Medicine, Cairo University, Giza, Egypt; 2 Pre-Treatment and Finishing of Cellulosic Fabric Department, Textile Research Division (TRD), National Research Centre (NRC), Dokki, Giza, Egypt; 3 Biochemistry and Chemistry of Nutrition Department, Faculty of Veterinary Medicine, Cairo University, Giza, Egypt; 4 PackingandPackaging Materials Department, National Research Center, Dokki, Giza, Egypt

**Keywords:** Polystyrene Nanoparticle, Kidneys, Histopathology, Malondialdehyde, Gene expression, COX-2

## Abstract

Polystyrene Nanoparticles (PS-NPs) used for packaging foam, disposable cups, and food containers. Therefore, this study aimed to evaluate PS- NPs toxic effects on kidney of adult male albino rats. A total of 30 rats divided into three groups (*n* = 10): group I negative control group; group II orally administered 3% PS-NPs (3 mg/kg body weight/day) and group III orally administered 3% PS-NPs (10 mg/kg body weight/day) for 35 days. Blood and kidney samples collected and processed for biochemical, histopathological, and immunohistochemical examinations. Results showed that low and high doses PS-NPs had significantly increased serum blood urea nitrogen (BUN), creatinine, malondialdehyde, significantly further reduced glutathione, downregulation of nuclear factor erythroid 2–related factor 2 and glutathione peroxidase, upregulation of caspase-3 and Cytochrome-c. Histopathological examination revealed several alterations. Low dose of PS-NPs exhibited dilated glomerular capillaries, hypotrophy of some renal corpuscles significantly decreases their diameter to 62 μm. Some proximal convoluted tubules and distal convoluted tubules showed loss of cellular architecture with pyknotic nuclei. Hyalinization and vacuolation in renal medulla. In high dose PS-NPs, alterations increased in severity. A significant increase in percentage area of cyclooxygenase-2 in low and high-doses. In conclusion, PS-NPs are a nephrotoxic causing renal dysfunction.

## Introduction

Plastic manufacturing has expanded over time (Li et al. 2016). Their products have become appropriate and cheap and are applied every day on all aspects of life (Geyer et al. 2017; Rhodes 2018). They are used as containers, plastic pressure pipe systems, plants pots, bottle caps, bags, netting, medical masks, industrial fibers, ropes, tanks and jugs, straws, appliances, centrifuge tubes, and car fenders(Ghosh et al. 2013; Li et al. 2016). Polymerization of several monomers with other substances forms artificial polymers of plastics (Thompson et al. 2009). The most widely used organic polymers in the plastic industry are polystyrene (PS), high-density polyethylene (HDPE), low-density polyethylene (LDPE), polyvinyl chloride (PVC), polyethylene terephthalate (PET), and polypropylene (PP) (Plastics – The Facts 2017). Plastics are very constantly used (Eriksen et al. 2014; Andrady 2017). About 79% of their wastes are accumulated in the natural environment, landfills, or dumps. Only 9% was recycled, and 12% was incinerated (Geyer et al. 2017). Plastics undergo fragmentation via photodegradation, abrasion with sand, contact with animals, the water itself, and erosion by wave action (Eriksen et al. 2014; Andrady 2017). Fragments can be classified into several kinds according to sizes: macroplastics and mesoplastics (diameter, > 5 mm) (Alimba and Faggio 2019), microplastics (MPs; diameter, 0.1–5 mm) (Andrady 2017; Karbalaei et al. 2018 and Alimba and Faggio 2019), and nanoplastics (NPs) (diameter, < 100 nm), which are produced through physical and biological degradation of microplastics by UV degradation (Yousif and Haddad 2013; Lambert and Wagner 2016).MPs/NPs are permanent in the environment (De Souza Machado et al. 2018; De Sá et al. 2018 and Alimba and Faggio 2019); thus, they pretend critical health and ecological attention (Akdogan and Guven, 2019). Moreover, they may play as vectors for chemical pollutants (Hartmann et al. 2017; Caruso 2019) and pathogens (Sgier et al. 2016; Wu et al. 2019). Polymerization of styrene monomers produces PS, a synthetic aromatic polymer. Styrene (vinylbenzene) is made from benzene and ethylene (Wünsch 2000) and has a chemical formula of (C8H8) n)(Ho et al. 2018). PS is broadly used in packaging foam, construction materials (insulation), disposable cups, food containers, cutleries, plates compact disks, and cassette boxes as it is relatively cheap and of good mechanical characteristics (Yang et al. 2015). Polystyrene nanoparticles (PS-NPs) have been extremely regarded as an example of nanoplastics to examine their toxicity and accumulation in organisms (Della Torre et al. 2014; Chen et al. 2017 and Rist et al. 2017). It is widely used in self-assembling nanostructures, biosensors, and photonics (Loss et al. 2014), drug delivery, personal care products, and bioimaging (Han et al. 2017; Wang et al. 2017). Microorganisms, plants, and animals are more severely affected by NPs than MPs, since they have a lesser diameter, assisting their permeation and accumulation in different tissues and organs (Mattsson et al. 2015). NP concentrations predicted in the environment are ≤ 1 μg/L (Lenz et al. 2016). NPs perhaps find their way to living organisms through food and water, air, and the skin. Thus, they can accumulate in different organs resulting in systemic exposure (Hernandez et al. 2017; Revel et al. 2018 and Yooeun et al. 2018). First, they will reach the intestinal epithelium that causes inflammation and viability failure (Wright and Kelly 2017). Then, translocation of PS-NPs from the gastrointestinal tract and consequently distributed throughout the body to the heart, kidney, etc. (Walczak et al. 2015b; Pitt et al. 2018a). NPs are suggested to enter the cell via pinocytosis, phagocytosis, or passive transport, and accordingly, they can enter the cellular membranes and several biological structures (Zhao et al. 2011; Shang et al. 2014). However, data on the mechanism of NP toxicity are still in its infancy. Proof for endocrine-disrupting effects of NP exposure is developed as they can interfere with the hormonal function and quantity (Halden 2010; Rochman et al. 2014 and Sussarellu et al. 2016). The common modes of action of endocrine-disrupting compounds are thyroid disruptors, androgen and estrogen antagonists and agonists, and aromatase inhibitors (Quintaneiro et al. 2017). Additionally, NPs cause cytotoxicity that provokes oxidative stress through free radicals generating from reactive oxygen species (ROS) (Barboza et al. 2018; Pitt et al. 2018b and Liu et al. 2019). They also evoke immunological responses (Brandts et al. 2018a; Revel et al. 2018), induce neurotoxicity (Barboza et al. 2018), stimulate genotoxicity (Brandts et al. 2018b; Jiang et al. 2019), and change the gene expression (Brandts et al. 2018b; Liu et al. 2019)aside from their reproductive and metabolic health effects (Rochman et al. 2014; Sussarellu et al. 2016).

Thusly, NPs produce several perils to wellbeing and climate as opposed to other plastic garbage. As far as we could possibly know, data on in vivo toxicity of NPs and their histopathological and biochemical modifications in rat species so far addresses the harmfulness on the base danger appraisal for people, while most examinations have zeroed in on the synthetic parts of NPs. To fill this hole, the current examination meant to assess the toxic impacts of ecologically important PS-NP focus as a NP model on the kidneys of adult male albino rats on histopathological, immunohistochemical, and biochemical levels.

## Materials and methods

### Chemicals

Styrene monomer (99.9%) was obtained from Sigma-Aldrich (USA). Therefore, styrene was distilled under reduced pressure before use. Tween 80 was purchased from Win Lab, India. Deionized water was used during preparation.

### Preparation of polystyrene nanoemulsion

First, PS microspheres were prepared. Second, the prepared PS microspheres were converted to nanoemulsion as follows: 250 mg of PS microspheres was dissolved in 100 mL of organic solvent; dimethyl sulfoxide (DMSO) and the mixture was stirred using a magnetic stirrer for 1 h. Then, an emulsifying agent (Tween 80 (200 mg)) was dissolved in 50 mL of deionized water using an ultrasonic homogenizer. At the end of dissolution for PS microspheres and Tween 80, the second solution (Tween 80) was added in drop-wise (at approximately 1 mL/s) to the first solution (PS microspheres) while stirring for another 1 h. After mixing and stirring, the organic solvent DMSO was drained using an evaporator. For further characterization and use, the prepared nanoemulsion was stored in the refrigerator.

#### Polystyrene characterization

The thermal stability of PS was determined using a Perkin Elmer thermal gravimetric analyzer (TGA) in an N_2_ atmosphere at a heating rate of 10 °C/min. A Vector-22 Fourier transform infrared spectrometer (FTIR) was used to record the PS’s infrared absorption spectra (Bruker Company, Germany).

### Experimental animals and ethical considerations

The study was carried out on 30 adult male albino rats weighing 225 ± 20 g. Rats were obtained from the breeding unit of the Animal Health Research Institute, Dokki, Egypt. They were housed at the Pharmacology Department in the Faculty of Veterinary Medicine in Cairo University. The rats were housed in plastic cages and handled for 2 weeks as an acclimatization period. They were fed standard food ad libitum and had access to water. Each rat was weighed once per week, and treatment doses were adjusted accordingly. Rats were kept at a constant temperature of 22 °C − 25 °C within a light-controlled room on an alternating 12:12-h light/dark cycle. The experimental protocol was approved by the Institutional Animal Care and Use Committee (IACUC) of the Faculty of Veterinary Medicine, Cairo University (protocol no. Vet CU 24,112,020,257).

### Experimental design

At the end of the acclimatization period, the rats were divided into three groups (*n* = 10 rats per group: 5 rats/cage). They were provided oral doses of the PS-NPs 3% via gavage needle for a 35-day experimental period as follows:

Group I (negative control group): received distilled water.

Group II: administered PS-NPs (3 mg/kg body weight/day) according to (Amereh et al. 2020).

Group III: administered PS-NPs (10 mg/kg body weight/day) according to (Amereh et al. 2020).

### Specimen and tissue preparation

All animals were anesthetized after 35 days, and blood samples were collected from the tail veins of all albino rats. These blood samples were collected in glass tubes and centrifuged at 3,000 rpm for 20 min; serum samples were stored at − 20 Cº until used to analyze kidney functions. Then, they were sacrificed through cervical decapitation within 30 min as per the ethical protocol approved by the IACUC at the Faculty of Veterinary Medicine, Cairo University. The kidneys were quickly removed. Some specimens were stored at − 80 ℃ for the measurement of oxidative stress parameters and Rt-PCR analysis. Other samples were fixed in 10% neutral-buffered formalin solution for histopathological and immunohistochemical examinations.

### Biochemical assay

#### Determination of kidney function markers

Blood urea nitrogen (BUN) was measured by urease colorimetric method and serum creatinine level was assayed using Buffered Kinetic jaffé reaction without deproteinization. The procedures were carried out according to reagent kits following the provided instructions (spectrum diagnostics. Egyptian Company for Biotechnology).

#### Renal oxidative stress biomarkers

Renal tissue was homogenized in an ice-cold 0.1-M phosphate-buffered saline (pH 7.4) using a Teflon tissue homogenizer. The crude tissue homogenate was centrifuged at 15,000 rpm for 15 min at 4 °C and used to measure malondialdehyde (MDA) according to Ohkawa et al. (1979), reduce glutathione (GSH) according to Ellman (1959), and measure total protein concentration according to the method described by Bradford (1976).

#### qRT-PCR analysis for *Nrf-2, GPX*, *Cytc*, and *CASP 3* genes

The relative renal *Nrf-2, GPx, Cytc,* and *CASP3* mRNA abundance was determined with qRT-PCR analysis using GAPDH as a housekeeping gene. Approximately, 100 mg renal tissue was used for total RNA extraction using the total RNA Extraction Kit (Vivantis, Malaysia). RT-PCR was performed using M-MuLV Reverse Transcriptase (NEB#M0253) after confirming the RNA concentration and purity. Quantitative assessment of cDNA amplification for each gene was performed using a fluorescence-based real-time detection method with a fluorescent SYBR Green dye (Thermo Scientific, Cat. No. K0221). The primer sequence used for qRT-PCR analysis is shown in Table (1) (Bashir et al. 2021; Hashim et al. 2021**)**. Real-time PCR conditions were performed as follows: 95 °C for 5 min and then 40 cycles at 95 °C for 15 s, 60 °C for 30 s, and 72 °C for 30 s. In each experiment, negative controls free of the template were included, and each qRT-PCR was performed with three biological replicates, and each biological replicate was assessed three times. The relative transcription level was calculated using the comparative 2^−ΔΔCT^ method (Livak and Schmittgen 2001**)**.

### Histopathological examination

#### Light microscope

Fixed samples were dehydrated with a series of alcohol washes followed by xylene and embedded in paraffin. Sections (3–4 μm thick) were prepared using a rotatory microtome. Then, tissue sections were deparaffinized and stained with hematoxylin and eosin (H&E) for histopathological examination (Bancroft and Gamble 2013).

#### Immunohistochemical examination

##### Cyclooxygenase 2 protein (COX-2)

A dark brown-colored stained cytoplasm is considered a positive response. About 5-µm-thick sections of the kidney were de-paraffinized and rehydrated; to retrieve antigen, sections were incubated with 0.1% trypsin and 0.1% CaCl2 2H2O in Tris buffer (50 mmol/l) at pH 7.4 at 371 °C for 120 min. Sections were soaked in absolute methanol containing 0.3% hydrogen peroxide for 30 min at room temperature to eliminate endogenous peroxidase activity. Then, sections were incubated with 1.5% non-immunized goat serum for 30 min at room temperature, incubated with diluted primary antibodies (1:500) for COX-2 for 30 min at room temperature, and washed three times using phosphate-buffered saline for 30 min. Thereafter, the sections were incubated with biotinylated goat antimouse immunoglobulin serum for 60 min, washed with phosphate-buffered saline, and incubated with the avidin/biotin peroxidase complex (Vector, Burlingame, California, USA). Sites of peroxidase binding were detected using chromogenic 3,30-diaminobenzidine tetrahydrochloride substrate. Tissue sections were counterstained with hematoxylin. The method used as outlined according to Côté et al. (1993).

#### Image analysis to evaluate immunohistochemical observations (area percentage)

Sections stained with anti-COX-2 were analyzed using a digital Leica Quin 500Â image analysis system (Leica Microsystems, Switzerland) housed at the Faculty of Dentistry, Cairo University. The image analyzer was automatically calibrated to convert pixels into units of area (μm^2^). COX-2 immunostaining was presented as a percentage of the total area in a standard measuring frame over ten independent fields from different slides in each group at 400 × magnification. All areas with positive immunohistochemical staining were evaluated, regardless of the intensity. The mean values and standard error (SE) obtained for each specimen were statistically analyzed.

### Statistical analysis

All quantitative results were analyzed using the SPSS version 17.0 for Windows. Data were presented as mean ± SE. Comparisons among multiple group means were performed using a one-way analysis of variance, followed by an LSD test. Statistical significance was set at p ≤ 0.05.

## Results

### Evaluation of the prepared polystyrene

**Fig. 1:**
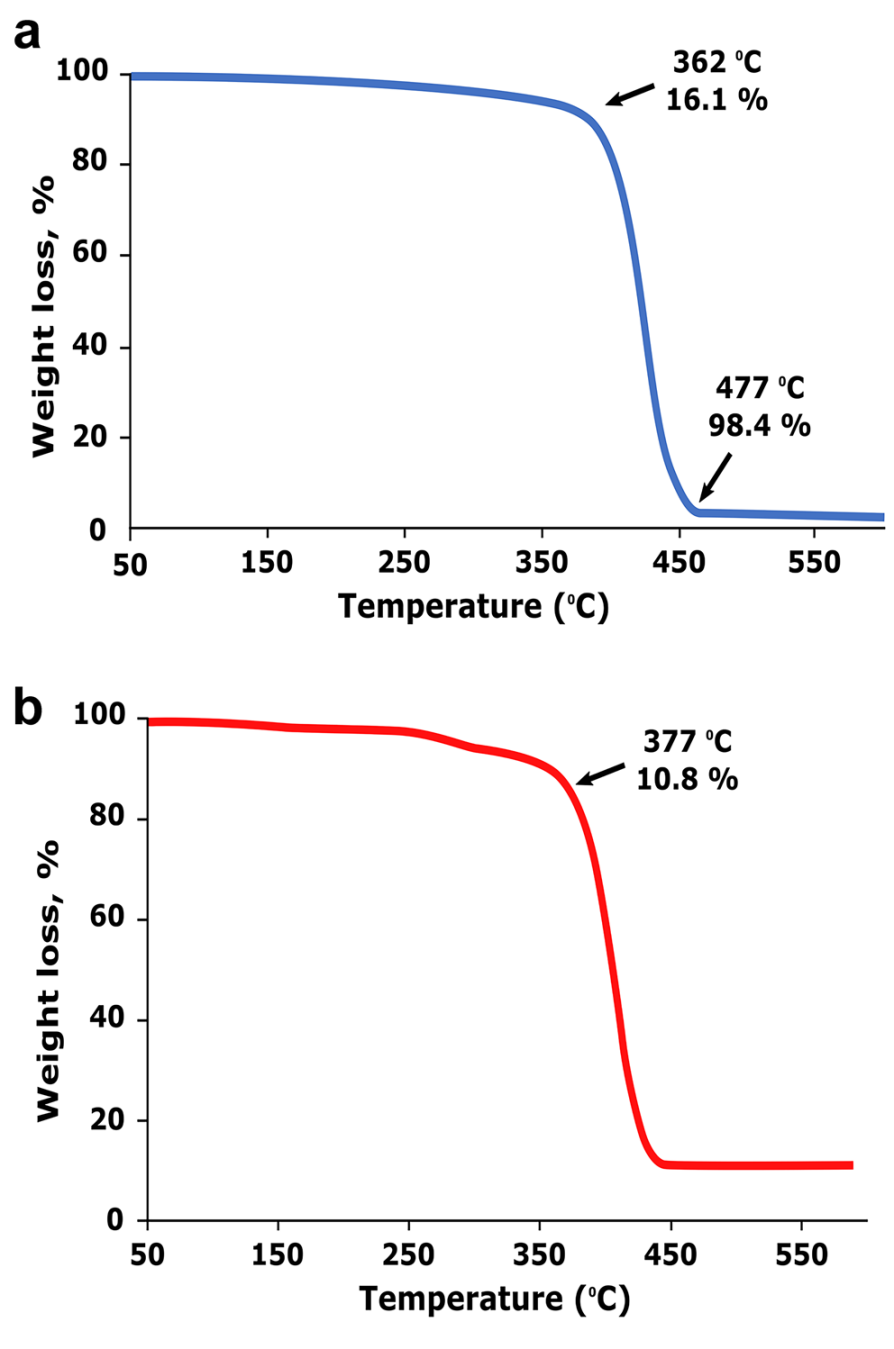
**(a)** TGA of Pure Polystyrene and **(b)** Polystyrene nanoemulsion

Thermal stability of the PS matrix was investigated using thermogravimetric analysis (TGA). PS microspheres and PS nanoemulsion TGA thermograms are shown in Fig. 1a and b. The thermal degradation structure of the PS microsphere was extracted from the TGA curve. Single-phase degradation was observed in PS microspheres due to decomposition of the PS matrix into volatile styrene monomers, which results in a mass loss of 98.4% at 411 °C. The evaporation of adsorbed water from the sulfonic acid groups resulted in the first weight loss from ambient temperature to 135 °C. The next zone was observed at approximately 411 °C, owing to the degradation of the sulfonic groups.

The thermal stability of PS nanoemulsion (Fig. 1b) is found to follow the order of degradation as shown with pure PS (Fig. 1a). However, the main two loss weight of PS nanoemulsion appeared at high temperatures when compared with pure PS. As observed in Fig. 1b that approximately 10.8% weight of PS nanoemulsion was lost at 377 °C and 88% of the weight has been lost at 411 °C. By comparing, the high thermal stability of PS nanoemulsion could be attributed to the high surface area, well stability, and small size of the produced nanoemulsion.

**Fig. 2:**
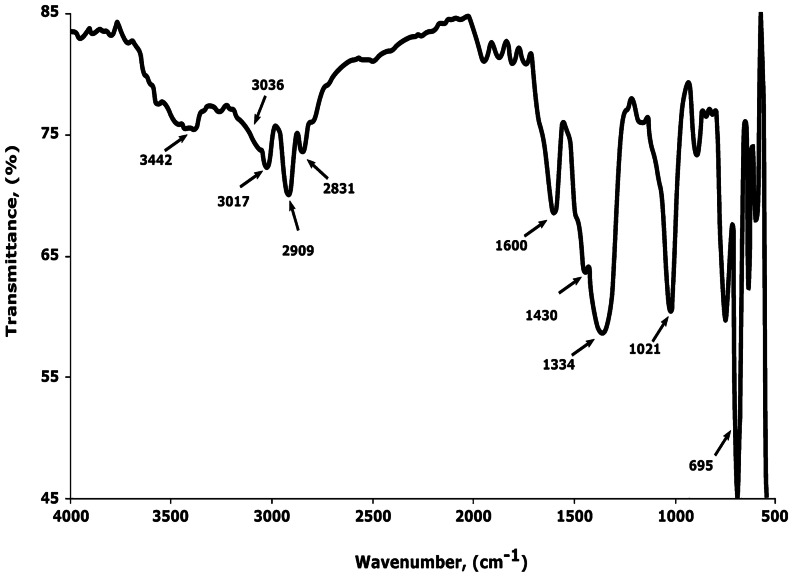
FTIR of the prepared Polystyrene nanoemulsion

Figure 2 shows the infrared absorption spectra (FTIR) of PS nanoemulsions. Numerous absorption peaks were observed within the wavenumber range in question. Because of aromatic C-H stretching vibration absorption, absorption peaks were observed at 3,036 and 3,017 cm^−1^, as well as aliphatic stretching at 2,909 and 2,831 cm^−1^, leading to the presence of methylenes.

Besides that, aromatic C = C stretching vibration absorption produces three absorption peaks at wavenumbers of 1,600, 1,430, and 1,334 cm^−1^. The existence of benzene rings is shown by these absorption peaks.

Furthermore, absorption peaks at 743 and 695 cm^−1^, which correlate to C-H out-of-plane bending vibration absorption, imply that the benzene ring contains only one substituent. Consequently, the stretching vibration absorption of O–H, which indicates the presence of hydroxyl, peaks at wavenumber 3,442 cm^−1^. Therefore, the difference in IR spectra of pure PS and polystyrene nanoemulsion is not different, implying that the nanoform reaction does not provide structure on the chemical structure of pure polystyrene.

#### Morphology of the prepared polystyrene

**Fig. 3:**
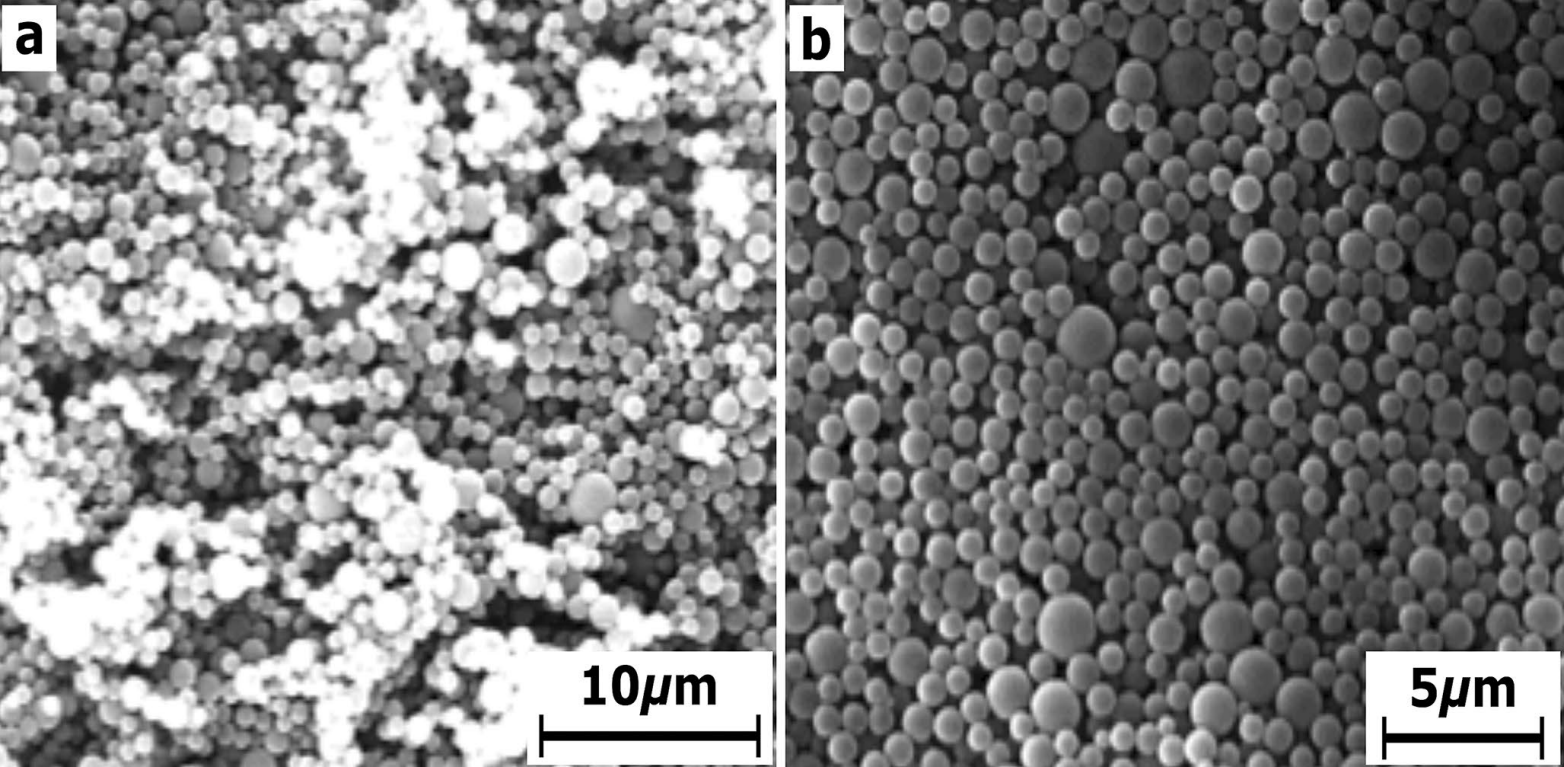
SEM image of the prepared polystyrene nanoemulsion at **(a)** low and **(b)** high magnification

Scanning electron microscopy (SEM) of the prepared PS nanoemulsion at low and high magnifications is displayed in Fig. 3a and 3b, showing the distinct form of the as-prepared PS nanoemulsion. The PS colloidal nanoemulsion is mostly arranged in a spherical form as exhibited from SEM images.

The colloidal crystal porosity of PS nanoemulsions with a spherical configuration is significantly higher than those with a tetragonal structure, which can be easily modified into a hexagonal configuration, a more compact structure that may have implications in medical applications.

### Biochemical investigations

#### Effect of PS-NPs on kidney function

**Fig. 4:**
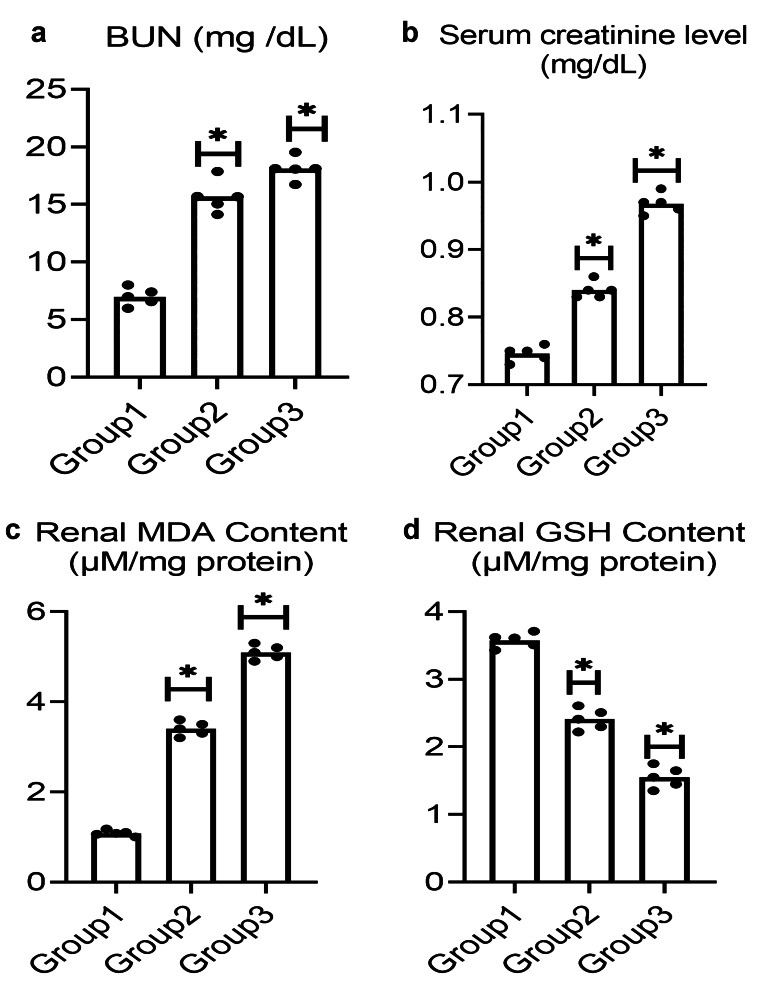
Effect of PS-NPs on (**a**) BUN (**b**) Serum creatinine (**c**) Renal MDA content and (**d**) Renal GSH content in male albino rat. Data are represented as mean ± SEM. * indicates significant difference from the corresponding control negative group at *p* ≤ 0.05

Renal damage was estimated using BUN and creatinine levels. Figure 4 reveals that a low dose of PS-NPs induced a significant increase in BUN levels from 6.98 to 15.69 mg/dL and serum creatinine level from 0.75 to 0.84 mg/dL when compared with the control group. Similarly, treatment with a high dose of PS-NPs also significantly elevated BUN and creatinine levels to 18.11 mg/dL and 0.97 mg/dL, respectively, when compared with the control group.

#### Oxidative stress biomarkers

##### A. MDA content in renal tissue

According to the obtained data in Fig. 4, treatment with low-dose PS-NPs significantly elevated the MDA renal content from 1.08 to 3.40 μΜ mg-1 protein when compared with the control group. Similarly, the high-dose treatment also elevated the MDA content to 5.10 μΜ mg-1 protein.

##### B. Renal content of GSH

Compared with the control group, renal GSH content was significantly reduced from 3.62 to 2.41 and to 1.55 μΜ mg-1 protein in rats treated with low and high doses of PS-NPs, respectively, as shown in Fig. 4.

#### qRT-PCR

##### A. qRT-PCR for some antioxidant-related genes (Nrf 2 and GPx)

**Fig. 5:**
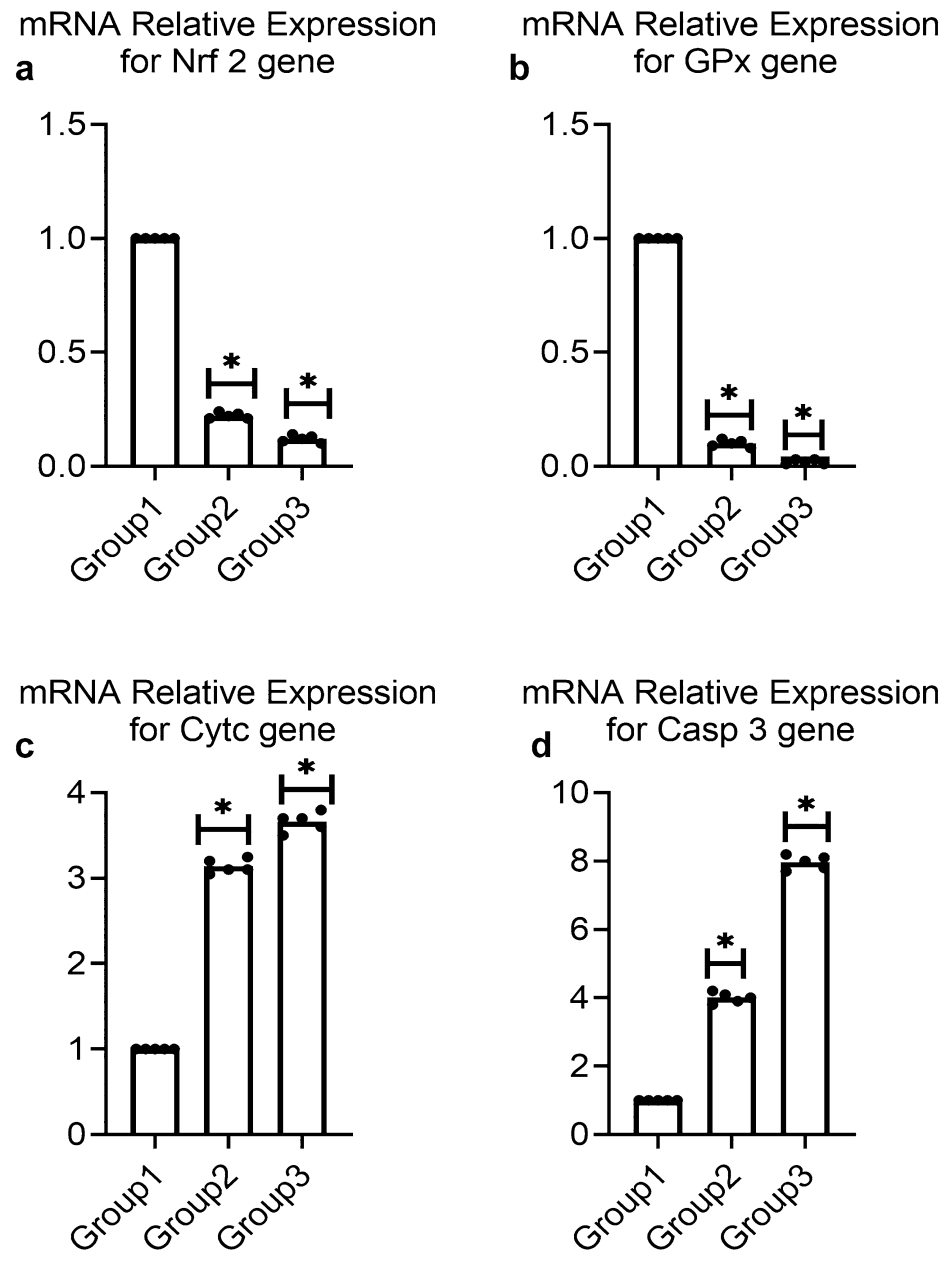
Effect of PS-NPs on renal mRNA relative expression for (**a**) *NRF-2* gene (**b**) *GPx* gene (**c**) *Cytc* gene and (**d**) *CASP 3* gene in male albino rat. Data are represented as mean ± SEM. * indicates significant difference from the corresponding control negative group at p ≤ 0.05

Contrasted to the control group, PS-NPs significantly diminished mRNA expression for Nrf-2 to 0.22-overlay and to 0.12-fold in the two groups treated with low and high doses, individually, as demonstrated in Fig. 5a. Besides, treatment with low and high doses demonstrated a significant diminishing in mRNA expression for GPX to 0.10-and 0.02-fold in comparison with the control group as demonstrated in Fig. 5b.

##### B. qRT-PCR of some apoptotic-related genes (*Cytc *and *CASP 3*)

Our current data revealed that treatment with both low and high doses of PS-NPs significantly increased the mRNA expression of *Cytc* to 3.10-fold and to 3.70-fold, respectively, when compared with the control. Moreover, CASP 3 gene expression was significantly elevated to 4.00-fold and to 8.00-fold in both low and high doses of PS-NPs, respectively, when compared with the control as shown in Fig. 5c and 5d.

### Histopathological examination

#### Light microscopy observations

**Fig.6:**
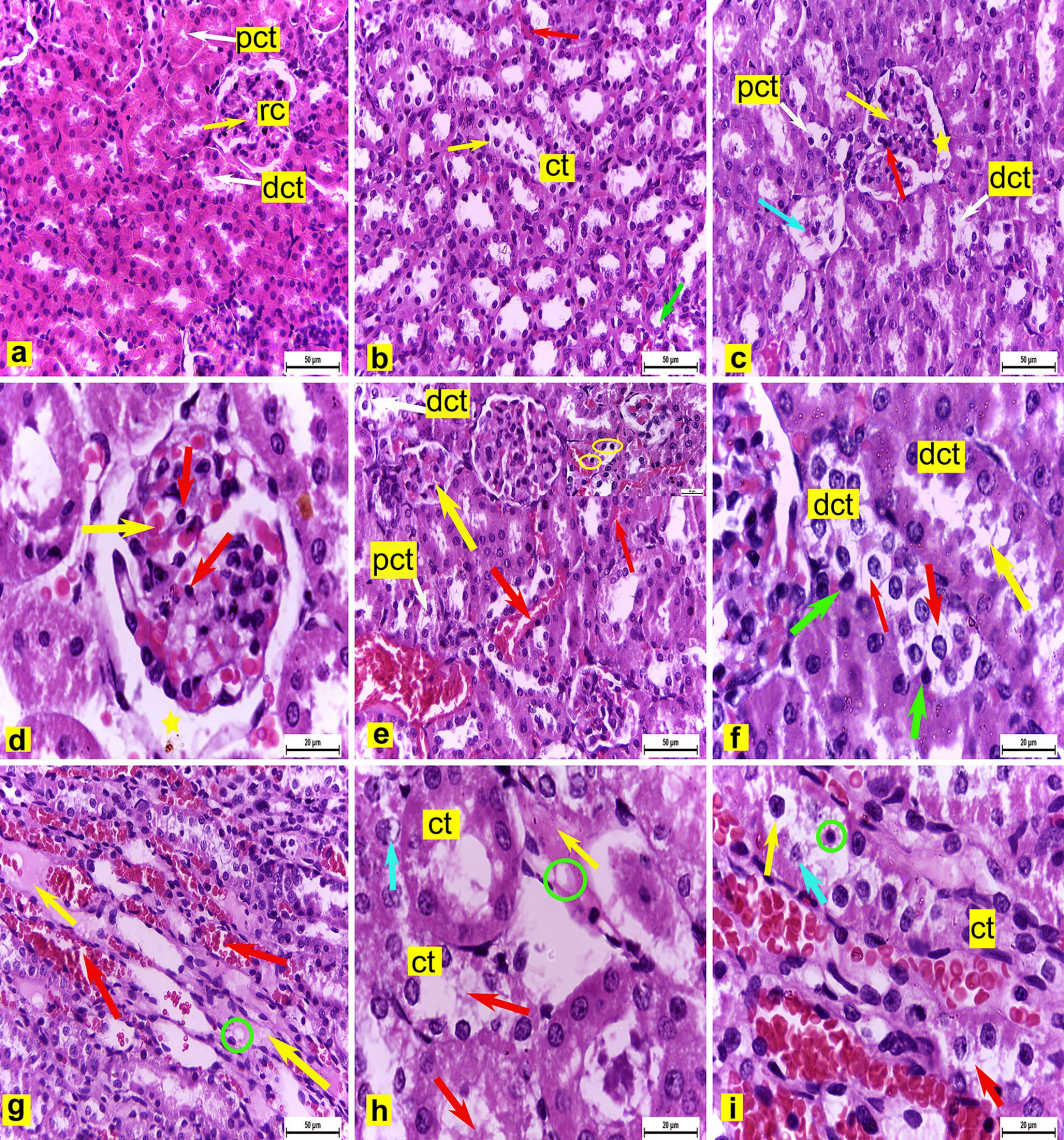
Renal tissue sections from adult albino male rats **(a: b)** Control rats (group I) showing **(a)** Renal cortex revealing normal histological structure, renal corpuscle (rc) containing glomerular capillaries (arrow), proximal convoluted tubule (pct), and distal convoluted tubule (dct). H&E X400. **(b)** Renal medulla containing collecting tubules (ct) lined by cuboidal cells (yellow arrow), loop of Henle's (green arrow), and blood capillaries (red arrow). H&E X400. **(c: i)** group II **(c:f)** Renal cortex showing **(c)** Dilated and engorged glomerular capillaries (yellow arrow), wide glomerular space (star), pyknotic nuclei of intraglomerular cells (red arrow), hypotrophied, degenerated renal corpuscle with loss of contents (blue arrow) and some (pct) and (dct) showed loss of the cellular architecture with pyknosis of their nuclei. H&E X400. **(d)** Dilated and engorged glomerular capillaries (yellow arrow), wide glomerular space (star), pyknotic nuclei of intraglomerular cells (red arrows) H&E X1000 **(e)** Hypotrophied renal corpuscle (yellow arrow), interstitial hemorrhage (red arrows), and some pct and dct lost their cellular architecture with pyknosis of some nuclei (inside cube X1000) H&E X400 **(f)** Some dct appeared with cytoplasmic strands in its lumen (yellow arrow) others demonstrated complete loss of the cytoplasmic acidophilia of their lining cells (red arrows)with pyknosis of some nuclei (green arrows).H&E X1000 **(g:i)** Renal medulla showing **(g)** Hyalinized areas (yellow arrows), vacuolation (circle), and dilatation and congestion of the interstitial blood capillaries (red arrows).H&E X400 **(h)** Hyalinized area (yellow arrow), vacuolation (circle), some degenerated collecting tubules (ct) with loss of the cellular cytoplasmic contents into their lumina (red arrows), and karyolitic nucleus of some tubular cells (blue arrow) H&E X1000 **(i)** Some collecting tubules (ct) cells partially lost their cytoplasmic acidophilia (red arrow) others completely lost it (yellow arrow), some nuclei appeared pyknotic and shrunken (circle) while others were karyolitic (blue arrow). H&E X1000

Assessment of H&E-stained kidney slides in the control group (Group I) of adult male albino rats showed ordinary histological structure where it was contained renal cortex and renal medulla. Renal cortex showed renal corpuscles with ordinary diameter containing glomerular capillaries and encompassed by Bowman's cases, proximal convoluted tubules (PCT) lined with pyramidal cells with restricted lumina, and distal convoluted tubules (DCT) lined with cuboidal cells with wide lumina (Fig. 6a). The renal medulla comprised of collecting tubules lined with cuboidal epithelia, loop of Henle's, and interstitial blood capillaries (Fig. 6b).

On the contrary, renal tissue sections of adult male albino rats from the experimental group II administered 3% PS-NPs (3 mg/kg bwt/day) revealed several histopathological alterations compared with the control group. The glomerular capillaries were dilated and engorged with blood with wide glomerular space and pyknosis in the nuclei of intraglomerular cells (Fig. 6c and d).Some renal corpuscles showed hypotrophy (Fig. 6c and e) and degeneration with content loss (Fig. 6c), with a significant decrease in their diameter to 62 μm Table (2) compared with the control group. Moreover, the renal cortex showed interstitial hemorrhage. Some PCT and DCT showed loss of the cellular architecture with pyknosis of their nuclei (Fig. 6c and e).Some DCT showed cellular cytoplasm shedding as strands in the tubular lumen, and others demonstrated complete loss of cytoplasmic acidophilia of their lining cellswith pyknosis of their nuclei(Fig. 6f).Additionally, the renal medulla showed some hyalinized areas with vacuolation (Fig. 6g and h) as well as dilatation and congestion of the interstitial blood capillaries(Fig. 6g).Some of the collecting tubules degenerated with loss of cellular cytoplasmic contents into their lumina (Fig. 6h).Some tubular cells partially lost their cytoplasmic acidophilia, whereas others completely lost it. Besides, some nuclei of the collecting tubule cells appeared pyknotic and shrunken (Fig. 6i), whereas others were karyolitic (Fig. 6h and i).

**Fig.7:**
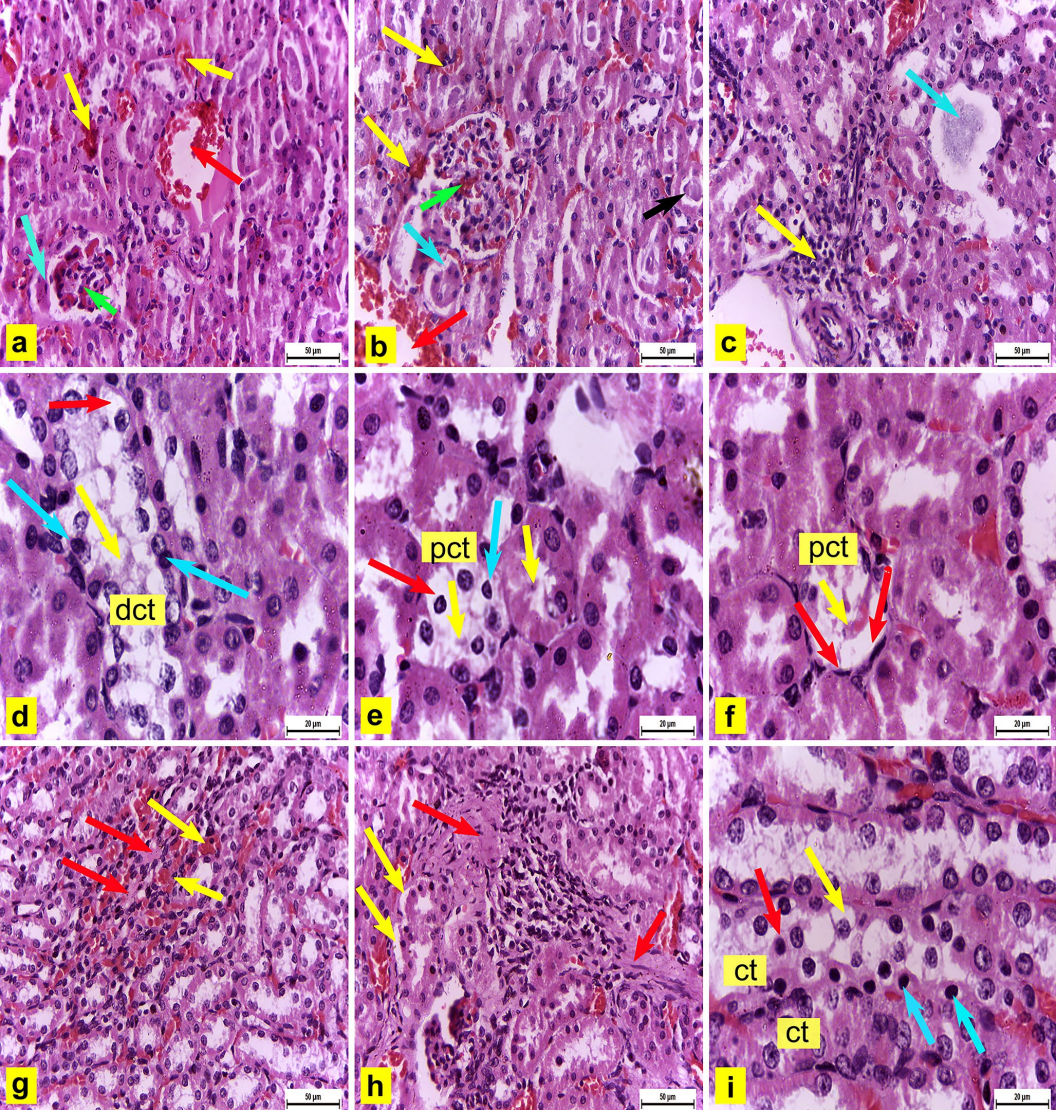
Renal tissue sections of adult albino male rats from group III **(a:f)** Renal cortex showing **(a)** Hemorrhage (yellow arrows), dilatation with congestion of the blood capillaries (red arrow) and glomerular capillaries (green arrow), and hypotrophied renal corpuscle (blue arrow) H&E X400. **(b)** Hemorrhage (yellow arrows), dilatation with congestion of the blood capillaries (red arrow) and glomerular capillaries (green arrow), accumulation of eosinophilic material (black arrow) in the tubular lumina**,** and peritubular space (blue arrow) H&E X400. **(c)** Hypotrophied, degenerated renal corpuscle with loss of contents (blue arrow) and mononuclear cell infiltration (yellow arrow) H&E X400 **(d)** Degenerated distal convoluted tubule (dct) with pyknosis of the nuclei (blue arrows), loss of the cytoplasmic acidophilia (red arrow) of their lining cells, and shedding of the cellular cytoplasm (yellow arrow) in the tubular lumen H&E X1000 **(e)** Degenerated proximal convoluted tubule (pct) with pyknosis of the nuclei (blue arrow), loss of the cytoplasmic acidophilia (red arrow)of their lining cells, and shedding of the cellular cytoplasm (yellow arrows) in the tubular lumina H&E X1000 **(f)** Degenerated (pct) with loss of cellular details, flattened pyknotic nuclei (red arrows), and shedding of cytoplasm in lumen (yellow arrow) H&E X1000. **(g:i)** Renal medulla showing **(g)** Interstitial hemorrhage (yellow arrows) and hyalinized patches (red arrows) H&E X400 **(h)** Fibrosis (red arrows) and peritubular spaces (yellow arrows) H&E X400 **(i)** Degenerated collecting tubules (ct) with loss of the cellular architecture, loss of cytoplasmic acidopilia (yellow arrow), shedding of cytoplasmic content and some nuclei into their lumina (red arrow), and pyknosis of some nuclei (blue arrows) H&E X1000

Conversely, renal tissue sections of adult male albino rats from the experimental group III administered 3% PS-NPs (10 mg/kg bwt/day) revealed an increased alteration severity observed in group II. The renal cortex showed hemorrhage, dilatation with congestion of blood capillaries and glomerular capillaries (Fig. 7a and b), and accumulation of eosinophilic material in the tubular lumina (Fig. 7b). Some renal corpuscles showed hypotrophy (Fig. 7a and c) and degeneration with complete content loss (Fig. 7c), with a significant decrease in their diameter to 59 μm compared with the control group but non-significant decrease as compared with group II Table (2). Peritubular spaces were observed in the cortex (Fig. 7b) with mononuclear cell infiltration (Fig. 7c). Some DCT and PCT showed degeneration, and their lining cells demonstrated pyknosis of their nuclei and loss of the cytoplasmic acidophilia. Some tubules showed shedding of the cellular cytoplasm in the tubular lumina (Fig. 7d and e). Other degenerated PCT showed loss of cellular details with flattened pyknotic nuclei and shedding of the cytoplasm in the lumina (Fig. 7f).

Consequently, the renal medulla showed interstitial hemorrhage and hyalinized patches (Fig. 7g). Obvious fibrosis and peritubular spaces were also observed (Fig. 7h). Some of the collecting tubules degenerated with loss of the cellular architecture, whereas others showed loss of their lining cells' cytoplasmic acidophilia, shedding of cytoplasmic content, and some nuclei into their lumina and pyknosis of some nuclei (Fig. 7i).

#### Immunohistochemical observations

**Fig. 8:**
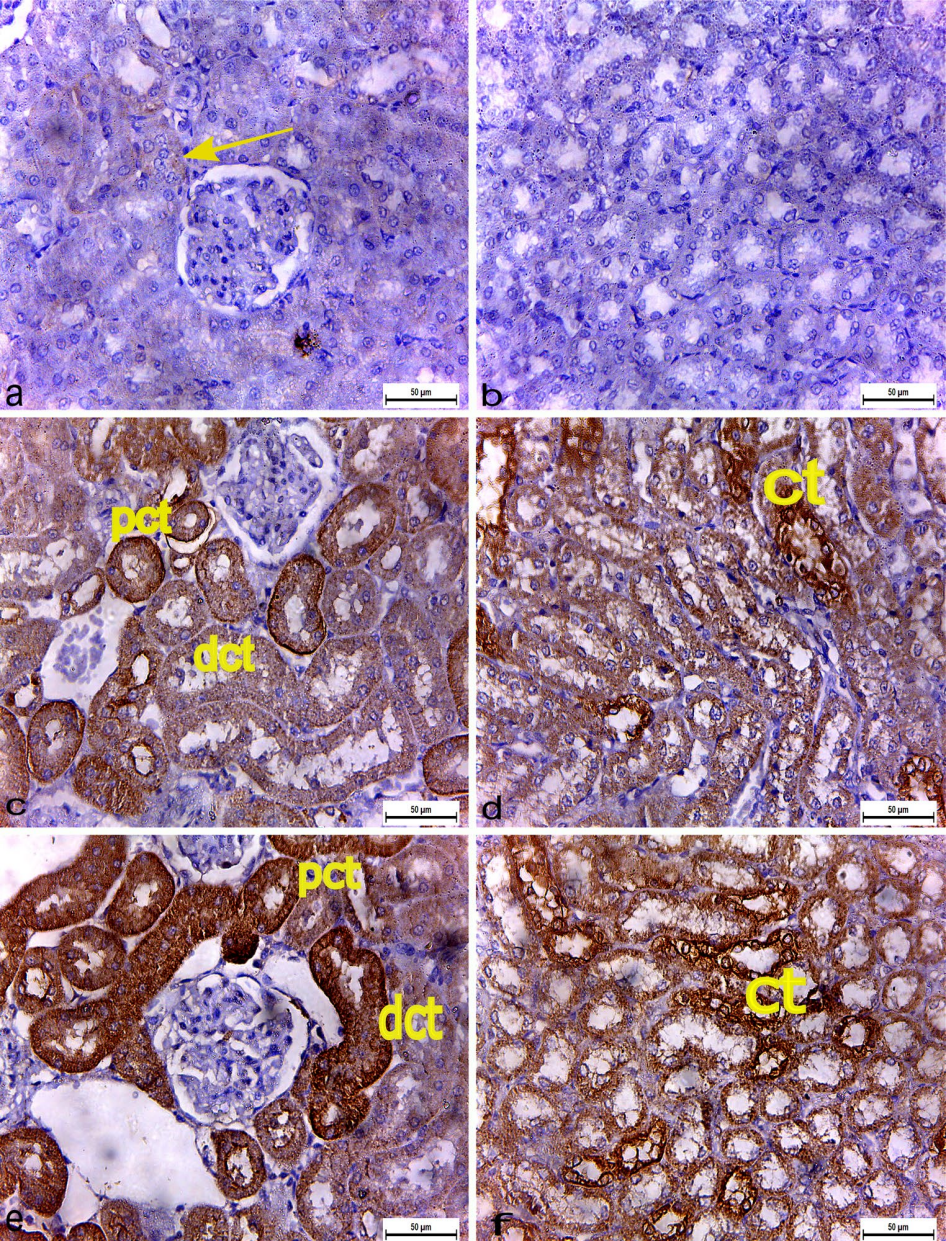
Immunohistochemically COX2 stained renal sections (X400) **(a)** Renal cortex **(b)** Renal medulla of control rats showing negligible cytoplasmic immunoexpression of COX-2 (arrow). **(c-d)** Group II 3% PS-NPs (3 mg/kg bwt) administered rats **(c)** Renal cortex showing positive COX-2 immunoreaction in cytoplasm of proximal convoluted tubules (pct) and distal convoluted tubules (dct) **(d**) Renal medulla showing positive immunoreaction in cytoplasm of collecting tubules' cells (ct). **(e–f**) Group III 3% PS-NPs (10 mg/ kg bwt) administered rats **(e)** Renal cortex showing strong positive COX-2 immunoexpression in cytoplasm of proximal convoluted tubules (pct) and distal convoluted tubules (dct) **(f)** Strong positive immunoreaction in cytoplasm of collecting tubules' cells (ct) in renal m

Immunohistochemical examination of both renal cortex and renal medulla of control rats (Group I) showed negligible cytoplasmic immunoexpression of COX-2 (Fig. 8a and b). Conversely, a positive immunoreactivity of COX-2 was observed in the cellular cytoplasm of PCT and DCT in the renal cortex and in the cytoplasm of collecting tubule cells in the renal medulla of rats administered 3% PS-NPs (3 mg/kg bwt) (GrouP II) as compared with the control group (Fig. 8c and d).Moreover, immunoexpression of COX-2 was strongly positive in the cellular cytoplasm of PCT and DCT in the renal cortex and in the cytoplasm of collecting tubules’ cells in the renal medulla of rats administered 3% PS-NPs (10 mg/kg bwt) (GrouP III) as compared with the control group (Fig. 8e and f).

**Fig. 9:**
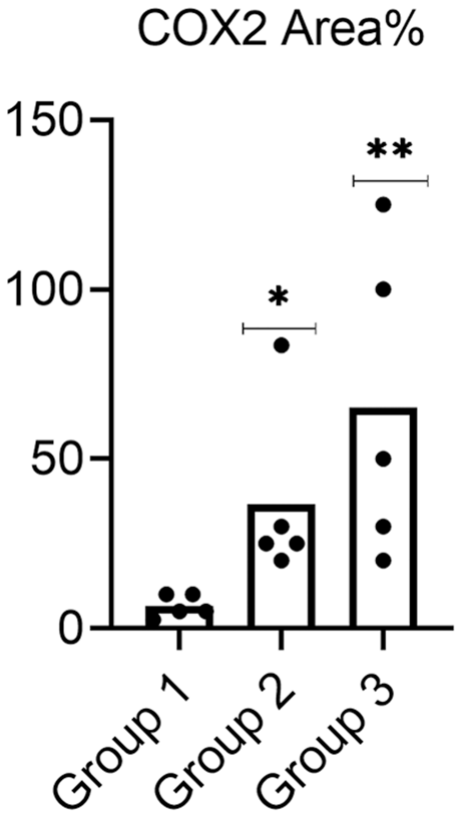
The effect of the two different doses (3 and 10 mg/kg bwt) of 3% PS-NPs on the percent area covered by COX-2-positive immunoreactive cells within the renal tissue of albino rats compared to control rats. Values are presented as mean ± SEM. *indicates a significant difference from the corresponding control negative group at *p* ≤ 0. 05. ** indicates a highly significant difference from the control group at *p *≤ 0.05

Data analysis showed a significant increase in the area% covered by COX-2-positive immunoreactive cells within the renal tissue in albino rats administered 3% PS-NPs (3 mg/kg bwt) (Group II) as compared to control rats to 36.7. Moreover, a highly significant increase in the area% covered by COX-2-positive immunoreactive cells was observed within the renal tissue in albino rats administered a high concentration of 3% PS-NPs (10 mg/kg bwt) (Group III) as compared to the control group to 65 (Fig. 9).

## Discussion

As of late, terms NPs have grabbed exceptional eye because of expanding openness levels of people. Among them, PS-NPs are quite possibly the most addressed NPs in the climate (Rubio et al. 2020). Curiously, fluorescent PS-NPs were demonstrated to be ingested by rodents and limited in the kidney tissue (Walczak et al. 2015a). This work intended to examine if and what PS-NPs mean for the kidney work and to investigate the fundamental likely instrument of its harmfulness in a rodent model. The rats were presented to various doses of PS-NPs for 35 days. Our outcomes showed that PS-NPs could dose conditionally incite nephrotoxicity as shown by significant elevation of renal biomarkers, increase in ROS production, and apoptosis. Significant elevation in serum creatinine and BUN shows renal dysfunction instigated by PS-NPs confirmed by a histopathological appraisal, and these discoveries are predictable with the investigation by Amereh et al. (2019). An increase in serum creatinine and BUN levels can reflect renal harm. BUN is viewed as the main intense renal marker that increments when any sort of injury happened in the kidney. Serum creatinine is a notable clinical pointer of kidney work and a more dependable marker of hindered glomerular filtration rate. The rise in kidney work biomarkers might be clarified by histological changes in the renal tissue, for the most part in the glomeruli and renal tubules. The renal tissue is exceptionally helpless to the improvement of oxidative abuses because of its long polyunsaturated unsaturated fat substance (Yin et al. 2019). The mechanism of PS-NP toxicity is a long way from being completely perceived, despite the fact that ROS has been proposed as the central participant in PS-NP harmfulness (Lehner et al. 2019). Specifically, exposure to NPs can initiate the ROS production (Liu et al. 2018). Besides, a few toxicological researches showed that oxidative pressure and ensuing DNA harm were related with toxicity incited by NPs (Prüst et al. 2020; Sarasamma et al. 2020). To analyze the oxidative stress reaction of PS-NPs on the renal tissue, MDA, GSH, GPx expression levels, and Nrf2 mRNA expression level were estimated in the current examination. A significant elevation of MDA was distinguished in the renal homogenate. Contrasted with the control, GSH and an expression for GPx and Nrf2 mRNA expression level are significantly lower in a dose-dependent way. This underscores the job of free radicals in oxidative cell damage because of PS-NPs harmfulness. Increased lipid peroxidation (LPO) is by and large considered as essential biomarkers for cell harms prompted by oxidative stress (Kim et al. 2013). MDA is utilized as a LPO index, and its height has been related with oxidative responses in various body tissues. A several examinations have recently connected increased MDA with renal injury in various models (Shi et al. 2017; Al Asmari et al. 2017). GSH is a significant endogenous nonenzymatic cell antioxidant molecule and is known to neutralize and scavenge a wide scope of ROS. GPx is a significant antioxidant enzyme for scavenging ROS and catalyzes the decrease of lipid peroxides. This capacity of GPx is fundamentally significant in antioxidant defense and support of the wellbeing of cells and creatures. In this investigation, expression of the gene encoding the GPx was clearly reduced by PS-NPs intoxication emphatically recommending that NPs could instigate oxidative harm. GSH and GPx consumption are related with the advancement of numerous cellular disturbances, including oxidative stress (Al-Olayan et al. 2016). Excessive ROS creation is known to assault film lipids and further upgrade their oxidation (Al-Brakati et al. 2019). Rubio et al. (2020) and Li et al. (2020) announced that PS-NPs can incite intracellular ROS creation and primary DNA harm, mirroring the part of oxidative stress in the mechanisms of PS-NPs toxicity (Hu and Palić. 2020). At the point when free radicals are overproduced, the body safeguards itself by combining enzymatic endogenous cell antioxidants or nonenzymatic ones (like GSH), which address the first line of protection against free radical harm (Hu and Palić. 2020). The large surface area and reactive nature of NPs give them gigantic oxidizing capacity (Rana et al. 2018). The molecular mechanism underlying PS-NPs' part in instigating renal oxidative harm isn't completely clear. Along these lines, Nrf2 mRNA gene expression was examined in the renal tissue. Momentarily, Nrf2 is a transcription factor accumulated in the nucleus in oxidative and electrophilic stress conditions, where it is considered as an expert controller for the expression of multiple antioxidant genes, like GSH, glutathione s transferase, superoxide dismutase enzyme, and catalase enzyme promote cell survival and help the cell to recover against oxidative stress (Manna et al. 2016).As indicated by the current information, PS-NP administration prompted a significant downregulation in the Nrf2 gene expression in a dose-dependent way in the renal tissue. As a significant component in cell number control, apoptosis is firmly connected with the most renal illness conditions (Sibarani et al. 2020). Renal apoptosis is related with the advancement of oxidative stress and inflammation, which in the long run prompted kidney injury (Yin et al. 2019). In the current examination, PS-NPs triggered apoptotic motioning by upregulating *casp* 3 and *Cytc* gene expression in the renal tissue. This is in accordance with a past report in which the authors reasoned that apoptosis assumes a fundamental part in PS-NP harmfulness (Jung et al. 2020). One of the vital components in renal apoptosis is caspase-3 (Jeruc et al. 2006; Ahmed et al. 2021). At the point when the sign advancing cell development vanishes, *Cytc* overexpression causes apoptosis (Rashad et al. 2018). Both intrinsic and extrinsic pathways of the apoptotic cycle can trigger the initiation of caspase 3. *Cytc* gene that emerges from mitochondrial harm instigates enactment of caspase-9, which thusly actuates the protein *casp 3*. *Casp 3* will be enacted in each cycle of renal cell apoptosis (Sibarani et al. 2020). Qu et al. (2019) and Chiu et al. (2015) revealed that PS-NPs initiated apoptosis and DNA harm through oxidative stress. The collection of PS-NPs in lysosomes could prompt the arrival of cathepsins into the cytosol, which eventually proliferated mitochondrial harm and therefore enacted cell apoptosis (Wang et al. 2013).

Nanoparticles (NPs) resulted in harmful cellular changes varying from acute cytotoxicity, neurotoxicity, and induction of inflammation to genotoxic effects (Nel et al. 2006). Nanoplastic particles interact with living organisms and cross biological barriers, are accumulated in organs, and affect cell functions (da Costa et al. 2016; Watts et al. 2016). Nanoplastic particles’ pathological mechanisms include the generation of ROS and induction of inflammation (Stone et al. 2007; Lu et al. 2016; Deng et al. 2017 and Prokić et al. 2019). Moreover, Walczak et al. (2015a, b) reported the PS-NP bioavailability and biodistribution from the gastrointestinal tract to various organs within 6 h. The maximum amounts of PS-NPs were calculated in the stomach and intestinal walls. PS-NPs were also found in the lung, spleen, testis, kidney, and heart, indicating that they exist systematically. Moreover, Babaei et al. (2020) concluded that dietary administration of PS-NPs was correlated with elevated circulating ROS concentrations in male Wistar rats. Therefore, the authors investigated the toxic impact of PS-NPs on the kidney of male adult albino rats as a target organ on histopathological and immunohistochemical levels.

In this study, renal tissue sections of adult male albino rats from experimental groups administered 3% PS-NPs in two different doses (3 mg and 10 mg/kg bwt/day) revealed several histopathological alterations as compared with the control group, such as dilation and engorgement of glomerular capillaries with blood, which agrees with the results of Abdelhalim and Jarrar (2011) in the renal cortex of Wistar rats intraperitoneally administered 10 nm to 20 nm of gold nanoparticles (GNPs) for 7 days. This dilatation might be due to the decreased vascular resistance of the renal tissue induced by PS-NPs. Furthermore, some renal corpuscles showed hypotrophy and degeneration with loss of their contents in both two doses but became more obvious in a group-administered higher dose of 3% PS-NPs (10 mg/kg bwt/day). This finding correlated to Ibrahim et al.’s (2018) study that reported diminished and distorted glomeruli of mice injected with 5 nm of GNPs. Histological alterations of renal corpuscles might suggest impaired renal function (Mohamed and Salah 2010). Interstitial hemorrhage was observed in the renal cortex and renal medulla, a finding consistent with that of Abdelhalim and Jarrar (2011).

Some PCT and DCT showed many alterations starting from loss of the cellular architecture, accumulation of eosinophilic material in the tubular lumina to end with degeneration in a higher dose of 3% PS-NPs (10 mg/kg bwt). These observations were suggested by Abdelhalim and Jarrar (2011) and Ibrahim et al. (2018). Moreover, DCT showed shedding of the cellular cytoplasm as strands in the tubular lumen. The same result was recorded by Abdelhalim and Jarrar (2011) who suggested GNPs affect a renal cell adhesion and induces cell–cell junction disruption. Moreover, cell–cell dissociation resulted from oxidative stress as mentioned by Inumaru et al. (2009), and PS-NPs induce the generation of ROS (Prokić et al. 2019), resulting in cell dissociation and shedding. Moreover, mononuclear cell infiltration observed in the renal cortex correlated with Abdelhalim and Jarrar (2011) and Ibrahim et al. (2018)’s findings. Johar et al. (2004) reported that GNPs could interact with proteins and enzymes of the renal interstitial tissue inhibiting the antioxidant defense mechanism and ROS generation, which may initiate an inflammatory response. In the current work, the renal medulla showed some hyalinized areas and degenerated collecting tubules. These degeneration steps result in necrosis that might be followed by organelle swelling, especially in the mitochondria, endoplasmic reticulum, and rupture of lysosomes before shrinking and dissolution of renal cell nuclei (Pandey et al. 2008). The severity of these lesions increased in the higher dose of 3% PS-NPs (10 mg/kg bwt).

Cyclooxygenase (COX) is an enzyme responsible for the formation of important biological mediators known as prostanoids, including prostaglandins, prostacyclin, and thromboxane. COX2 is undetectable in most normal tissues and is an inducible enzyme in most tissues exposed to inflammation (Warner and Mitchell, 2002). In our research, immunohistochemical examination revealed positive COX-2 immunoexpression in the cellular cytoplasm of PCT and DCT in the renal cortex and in the cytoplasm of collecting tubules’ cells in the renal medulla of rats administered the low dose of 3% PS-NPs (3 mg/kg bwt), which showed a significantly strong positive immunoreaction in a high dose of 3% PS-NPs (10 mg/kg bwt). These results suggest that PS-NPs induce apoptosis, a finding consistent with that of Qu et al. (2019) who reported that PS-NPs induced apoptosis and DNA damage through oxidative stress. The histological and the histochemical alterations in the renal tissues induced by PS -NPs could be documented the accumulation of PS-NPs in renal tissue. The induced histological alterations might be an indication of injured renal tubules due to PS-NPs toxicity that caused accumulated residues resulting from metabolic and structural disturbances in renal tissue.

## Conclusion

The use of plastics has been extremely increased every day due to their low cost and appropriate characteristics. Thus, these plastics are degraded and fragmented into small particles (MPs/NPs), which are increasingly accumulated in the environment and causing health hazards. Our findings demonstrated the PS-NP toxicity on the kidneys of rats (as models for mammals), the adversely affected species. Renal function impairment and several histopathological alterations were recorded as a consequence of the oxidative damage and apoptosis-induced PS-NP toxicity. However, several concentrations are one of the limitations of this study, and thus, sizes of these particles greatly varied in different studies. Additionally, exposure levels in humans remain unknown and will be different from the rat model. Therefore, we recommend performing further investigations to fill the gap in this aspect.

**Table 1:** Primer sequence used for qRT-PCR

Gene symbol	Gene description	Accession number	Primer Sequence
*GAPDH*	Glyceraldehyde3-phosphate dehydrogenase	NC_005103.4	F:- 5′-ACCACAGTCCATGCCATCAC-3′ R:- 5′-TCCACCACCCTGTTGCTGTA-3′
*Nrf 2*	Nuclear factor, erythroid 2-like 2	NC_005102.4	F: -5′‐GGCCCTCAATAGTGCTCAG‐3′ R:-5′‐TAGGCACCTGTGGCAGATTC‐3′
*GPX*	Glutathione peroxidase	M21210.1	F:-5′-CTCTCCGCGGTGGCACAGT-3′ R:- 5-CCACCACCGGGTCGGACATAC‐3′
*Cytc*	Cytochrome c	K00750.1	F:- 5′-TAC CC T CTC AAC GAC AGC AG-3′ R:- 5′-TCT TGA CAT TCT CCT CGG TG-3′
*CASP 3*	Caspase 3	NM_012922.2	F: -5ʹ-GGAGCTTGGAACGCGAAGAA-3ʹ R:-5ʹ-ACACAAGCCCATTTCAGGGT-3ʹ

**Table 2:** The effect of the two different doses (3 and 10 mg / kg bwt/day)of 3% PS-NPs on the diameter of the glomerulus in the renal tissues of albino rats compared to control albino rats

Groups	Diameter of glomerulus (µm)
GrouP I (control)	78 ± 2
GrouP II (3 mg/kg bwt)	62 ± 0.6*
GrouP III (10 mg/kg bwt)	59 ± 1.4*

Values are presented as mean ± SEM. *indicates significant difference from the corresponding control negative group at p ≤ 0. 05

## Data Availability

Not applicable.

## References

[CR1] Abdelhalim MAK, Jarrar BM (2011). The appearance of renal cells cytoplasmic degeneration and nuclear destruction might be an indication of GNPs toxicity. Lipids Health Dis.

[CR2] Ahmed ZSO, Galal MK, Drweesh EA, Abou-El-Sherbini KS, Elzahany EA, Elnagar MM, Yasin NA (2021). Protective effect of starch-stabilized selenium nanoparticles against melamine-induced hepato-renal toxicity in male albino rats. Int J Biol Macromol.

[CR3] Akdogan Z, Guven B (2019) Microplastics in the environment: A critical review of current understanding and identification of future research needs. Environ Pollut 254, 113011. 10.1016/j.envpol.2019.113011 31404735

[CR4] Alimba CG, Faggio C (2019). Microplastics in the marine environment: Current trends in environmental pollution and mechanisms of toxicological profile. Environ Toxicol Pharmacol.

[CR5] Al Asmari AK, Al Sadoon KT, Obaid AA, Yesunayagam D, Tariq M (2017) Protective effect of quinacrine against glycerol-induced acute kidney injury in rats. BMC nephrology 18(1):1–10. 10.1186/s12882-017-0450-8 PMC5273840 28129740

[CR6] Al-Brakati AY, Kassab RB, Lokman MS, Elmahallawy EK, Amin HK, Abdel Moneim AE (2019). Role of thymoquinone and ebselen in the prevention of sodium arsenite–induced nephrotoxicity in female rats. Hum Exp Toxicol.

[CR7] Al-Olayan EM, El-Khadragy MF, Omer SA, Shata MTM, Kassab RB, Abdel Moneim AE (2016) The beneficial effect of cape gooseberry juice on carbon tetrachloride-induced neuronal damage. CNS & Neurol Disorders-Drug Targets (Formerly Current Drug Targets-CNS & Neurological Disorders) 15(3):344–350. 10.2174/1871527314666150821112051 26295813

[CR8] Amereh F, Eslami A, Fazelipour S, Rafiee M, Zibaii MI, Babaei M (2019). Thyroid endocrine status and biochemical stress responses in adult male Wistar rats chronically exposed to pristine polystyrene nanoplastics. Toxicology Research.

[CR9] Amereh F, Babaei M, Eslami A, Fazelipour S, Rafiee M (2020) The emerging risk of exposure to nano (micro) plastics on endocrine disturbance and reproductive toxicity: From a hypothetical scenario to a global public health challenge. Environ Pollut 261:114158. 10.1016/j.envpol.2020.114158 32088433

[CR10] Andrady AL (2017). The plastic in microplastics: a review. Mar Pollut Bull.

[CR11] Babaei AA, Rafiee M, Khodagholi F, Ahmadpour E, Amereh F (2020) Physiological stress response of the Wistar albino rats orally exposed to polystyrene nanoparticles. Part Fib Toxic J.

[CR12] Bashir DW, Rashad MM, Ahmed YH, Drweesh EA, Elzahany EA, Abou-El-Sherbini KS, Ebtihal MEL (2021) The ameliorative effect of nanoselenium on histopathological and biochemical alterations induced by melamine toxicity on the brain of adult male albino rats. NeuroToxicology. 10.1016/j.neuro.2021.06.006 34216684

[CR13] Bancroft JD, Gamble M (2013) Theory and practice of histological techniques, Churchill Livingstone 173–179.

[CR14] Barboza LGA, Vieira LR, Branco V, Figueiredo N, Carvalho F, Carvalho C, Guilhermino L (2018). Microplastics cause neurotoxicity, oxidative damage and energy-related changes and interact with the bioaccumulation of mercury in the European seabass, Dicentrarchuslabrax (Linnaeus, 1758). Aquat Toxicol.

[CR15] Bradford MM (1976). A rapid and sensitive method for the quantitation of microgram quantities of protein utilizing the principle of protein-dye binding. Anal Biochem.

[CR16] Brandts I, Teles M, Goncalves AP, Barreto A, Franco-Martinez L, Tvarijonaviciute A, Martins MA, Soares A, Tort L, Oliveira M (2018). Effects of nanoplastics on Mytilus galloprovincialis after individual and combined exposure with carbamazepine. Sci Total Environ.

[CR17] Brandts I, Teles M, Tvarijonaviciute A, Pereira ML, Martins MA, Tort L, Oliveira M (2018). Effects of polymethylmethacrylate nanoplastics on Dicentrarchuslabrax. Genomics.

[CR18] Caruso G (2019). Microplastics as vectors of contaminants. Mar Pollut Bull.

[CR19] Chen Q, Gundlach M, Yang S, Jiang J, Velki M, Yin D, Hollert H (2017). Quantitative investigation of the mechanisms of microplastics and nanoplastics toward zebrafish larvae locomotor activity. Sci Total Environ.

[CR20] Chiu HW, Xia T, Lee YH, Chen CW, Tsai JC, Wang YJ (2015). Cationic polystyrene nanospheres induce autophagic cell death through the induction of endoplasmic reticulum stress. Nanoscale.

[CR21] Côté A, da Silva R, Cuello AC (1993) Current protocols for light microscopy immunocytochemistry. In: Cuello AC, editor. Immunohistochemistry II: John Wiley & Sons, Chichester 147–168.

[CR22] da Costa JP, Santos PS, Duarte AC, Rocha-Santos T (2016). Nano plastics in the environment–sources, fates and effects. Sci Total Environ.

[CR23] Della Torre C, Bergami E, Salvati A, Faleri C, Cirino P, Dawson KA, Corsi I (2014). Accumulation and embryotoxicity of polystyrene nanoparticles at early stage of development of sea urchin embryos Paracentrotuslividus. Environ Sci Technol.

[CR24] Deng Y, Zhang Y, Lemos B, Ren H (2017). Tissue accumulation of microplastics in mice and biomarker responses suggest widespread health risks of exposure. Sci Rep.

[CR25] De Sá LC, Oliveira M, Ribeiro F, Rocha TL, Futter MN (2018). Studies of the effects of microplastics on aquatic organisms: What do we know and where should we focus our efforts on the future?. Sci Total Environ.

[CR26] De Souza Machado AA, Kloas W, Zarfl C, Hempel S, Rillig MC (2018). Microplastics as an emerging threat to terrestrial ecosystems. Glob Chang Biol.

[CR27] Ellman GE (1959). Tissue Sulphydryl Groups Arch Biochem Biophys.

[CR28] Eriksen M, Lebreton LCM, Carson HS, Thiel M, Moore CJ, Borerro JC (2014). Plastic pollution in the world’s oceans: more than 5 trillion plastic pieces weighing over 250,000 tons afloat at sea. PLoS ONE.

[CR29] Geyer R, Jambeck JR, Law KL (2017) Production, use, and fate of all plastics ever made. Sci Adv 3:e1700782. 10.1126/sciadv.1700782 PMC5517107 28776036

[CR30] Ghosh SK, Pal S, Ray S (2013). Study of microbes having potentiality for biodegradation of plastics. Environ Sci Pollut Res Int.

[CR31] Halden RU (2010) Plastics and health risks. Annu Rev Publ Health 31:179e194. 10.1146/annurev.publhealth.012809.103714 20070188

[CR32] Han B, Liu W, Li J, Wang J, Zhao D, Xu R, Lin Z (2017). Water Res.

[CR33] Hartmann NB, Rist S, Bodin J, Jensen LH, Schmidt SN, Mayer P, Meibom A, Baun A (2017). Microplastics as vectors for environmental contaminants: Exploring sorption, desorption, and transfer to biota. Integr Environ Assess Manag.

[CR34] Hashim AR, Bashir DW, Yasin NA, Galal MK, El-Gharbawy SM (2021) Ameliorative effect of N-acetylcysteine against glyphosate-induced hepatotoxicity in adult male albino rats: histopathological, biochemical, and molecular studies. Env Sci Pollut Res 1–15. 10.1007/s11356-021-13659-2 33797725

[CR35] Hernandez LM, Yousefi N, Tufenkji N (2017) Are there nanoplastics in your personal care products? Environ Sci Technol Lett 4(7):280e285.

[CR36] Ho BT, Roberts TK, Lucas S (2018). An overview on biodegradation of polystyrene and modified polystyrene: the microbial approach. Crit Rev Biotechnol.

[CR37] Hu M and Palić D (2020) Micro-and nano-plastics activation of oxidative and inflammatory adverse outcome pathways. Redox Biology 101620**.** 10.1016/j.redox.2020.101620 PMC7767742 32863185

[CR38] Ibrahim KE, Al-Mutary MG, Bakhiet AO, Khan HA (2018). Histopathology of the liver, kidney, and spleen of mice exposed to gold nanoparticles. Molecules.

[CR39] Inumaru J, Nagano O, Takahashi E, Ishimoto T, Nakamura S, Suzuki Y, Niwa SI, Umezawa K, Tanihara H, Saya H (2009). Molecular mechanisms regulating dissociation of cell–cell junction of epithelial cells by oxidative stress. Genes Cells.

[CR40] Jeruc J, Vizjak A, Rozman B, Ferluga D (2006). Immunohistochemical expression of activated caspase-3 as a marker of apoptosis in glomeruli of human lupus nephritis. Am J Kidney Dis.

[CR41] Jiang X, Chen H, Liao Y, Ye Z, Li M, Klobucar G (2019). Ecotoxicity and genotoxicity of polystyrene microplastics on higher plant Viciafaba. Environ Pollut.

[CR42] Johar D, Roth JC, Bay GH, Walker JN, Kroczak TJ, Los M (2004) Inflammatory response, reactive oxygen species, programmed (necrotic-like and apoptotic) cell death and cancer. 15631311

[CR43] Jung BK, Han SW, Park SH, Bae JS, Choi J, Ryu KY (2020). Neurotoxic potential of polystyrene nanoplastics in primary cells originating from mouse brain. Neurotoxicology.

[CR44] Karbalaei S, Hanachi P, Walker TR, Cole M (2018). Occurrence, sources, human health impacts and mitigation of microplastic pollution. Environ Sci Pollut Res.

[CR45] Kim SW, Kwak JI, An YJ (2013). Multigenerational study of gold nanoparticles in Caenorhabditis elegans: transgenerational effect of maternal exposure. Environ Sci Technol.

[CR46] Lambert S and Wagner M (2016) Characterisation of nanoplastics during the degradation of polystyrene. Chemosphere 145, 265–268. 10.1016/j.chemosphere.2015.11.078 PMC5250697 26688263

[CR47] Lehner R, Weder C, Petri-Fink A, Rothen-Rutishauser B (2019). Emergence of nanoplastic in the environment and possible impact on human health. Environ Sci Technol.

[CR48] Lenz R, Enders K, Nielsen TG (2016). Microplastic exposure studies should be environmentally realistic. Proc Natl Acad Sci USA.

[CR49] Liu Z, Cai M, Yu P, Chen M, Wu D, Zhang M, Zhao Y (2018). Age-dependent survival, stress defense, and AMPK in Daphnia pulex after short-term exposure to a polystyrene nanoplastic. Aquat Toxicol.

[CR50] Liu Z, Yu P, Cai M, Wu D, Zhang M, Huang Y, Zhao Y (2019). Polystyrene nanoplastic exposure induces immobilization, reproduction, and stress defense in the freshwater cladoceran Daphnia pulex. Chemosphere.

[CR51] Livak KJ and Schmittgen TD (2001) Analysis of relative gene expression data using real-time quantitative PCR and the 2− ΔΔCT method. methods, 25(4), 402–408. 10.1006/meth.2001.1262 11846609

[CR52] Li WC, Tse HF, Fok L (2016). Plastic waste in the marine environment: A review of sources, occurrence and effects. Sci Total Environ.

[CR53] Li X, Hu J, Qiu R, Zhang X, Chen Y, He D (2020). Joint toxic effects of polystyrene nanoparticles and organochlorine pesticides (chlordane and hexachlorocyclohexane) on Caenorhabditis elegans. Environ Sci Nano.

[CR54] Loss C, Syrovets T, Musyanovych A, Mailander V, Landfester K, Nienhaus UG, Simmet T (2014) Functionalized polystyrene nanoparticles as a platform for studying bio-nano interactions. Beilstein J Nanotechnol 5:2403e2412. 10.3762/bjnano.5.250 PMC4311717 25671136

[CR55] Lu Y, Zhang Y, Deng Y, Jiang W, Zhao Y, Geng J, Ding L, Ren H (2016). Uptake and accumulation of polystyrene microplastics in zebrafish (Danio rerio) and toxic effects in liver. Environ Sci Technol.

[CR56] Manna K, Khan A, Biswas S, Das U, Sengupta A, Mukherjee D, Chakraborty A, Dey S (2016). Naringin ameliorates radiation-induced hepatic damage through modulation of Nrf2 and NF-κB pathways. RSC Adv.

[CR57] Mattsson K, Hansson LA, Cedervall T (2015) Nano-plastics in the aquatic environment. Environ Sci Process Impacts 17(10):1712e1721. 10.1039/c5em00227c 26337600

[CR58] Mohamed NA, Saleh SM (2010). Effect of Pre and Postnatal Exposure to Lead Acetate on the Kidney of Male Albino Rat: A Light and Electron Microscopic Study. Egypt J Histol.

[CR59] Nel A, Xia T, Mädler L, Li N (2006) Toxic potential of materials at the nanolevel. science, 311(5761), pp.622–627. 10.1126/science.1114397 16456071

[CR60] Ohkawa H, Ohishi N, Yagi K (1979). Assay for lipid peroxides in animal tissues by thiobarbituric acid reaction. Anal Biochem.

[CR61] Pandey G, Srivastava DN, Madhuri S (2008). A standard hepatotoxic model produced by paracetamol in rat. Toxicol Int.

[CR62] Pitt JA, Kozal JS, Jayasundara N, Massarsky A, Trevisan R, Geitner N, Wiesner M, Levin ED, Di Giulio RT (2018). Uptake, tissue distribution, and toxicity of polystyrene nanoparticles in developing zebrafish (Danio rerio). Aquat Toxicol.

[CR63] Pitt JA, Trevisan R, Massarsky A, Kozal JS, Levin ED, Di Giulio RT (2018). Maternal transfer of nanoplastics to o_spring in zebrafish (Danio rerio): A case study with nanopolystyrene. Sci Total Environ.

[CR64] Plastics-The Facts (2017) An Analysis of European Plastics Production, Demand and Waste Data. https://www.plasticseurope.org/application/files/5715/1717/4180/Plastics_the_facts_2017_FINAL_for_website_one_page.pdf.

[CR65] Prokić MD, Radovanović TB, Gavrić JP, Faggio C (2019). Ecotoxicological effects of microplastics: Examination of biomarkers, current state and future perspectives. TrAC, Trends Anal Chem.

[CR66] Prüst M, Meijer J, Westerink RH (2020). The plastic brain: neurotoxicity of micro-and nanoplastics. Part Fibre Toxicol.

[CR67] Quintaneiro C, Patrício D, Novais S, Soares A, Monteiro M (2017). Endocrine and physiological effects of linuron and S-metolachlor in zebrafish developing embryos. Sci Total Environ.

[CR68] Qu M, Qiu Y, Kong Y, Wang D (2019) Amino modification enhances reproductive toxicity of nanopolystyrene on gonad development and reproductive capacity in nematode Caenorhabditis elegans. Environ Pollut 254:112978. 10.1016/j.envpol.2019.112978 31398636

[CR69] Rana K, Verma Y, Rani V, Rana SVS (2018). Renal toxicity of nanoparticles of cadmium sulphide in rat. Chemosphere.

[CR70] Rashad MM, Galal MK, EL-Behairy, A. M., Gouda, E. M. & Moussa, S. Z.  (2018). Maternal exposure to di-n-butyl phthalate induces alterations of c-Myc gene, some apoptotic and growth related genes in pups’ testes. Toxicol Ind Health.

[CR71] Revel M, Yakovenko N, Caley T, Guillet C, Chatel A, Mouneyrac C (2018) Accumulation and immunotoxicity of microplastics in the estuarine worm Hedistediversicolor in environmentally relevant conditions of exposure. Environ Sci Pollut Res Int. 10.1007/s11356-018-3497-6 30353435

[CR72] Rhodes CJ (2018). Plastic pollution and potential solutions. Sci Prog.

[CR73] Rist S, Baun A, Hartmann NB (2017). Ingestion of micro- and nanoplastics in Daphnia magna – quantification of body burdens and assessment of feeding rates and reproduction. Environ Pollut.

[CR74] Rochman CM, Kurobe T, Flores I, Teh SJ (2014). Early warning signs of endocrine disruption in adult fish from the ingestion of polyethylene with and without sorbed chemical pollutants from the marine environment. Sci Total Environ.

[CR75] Rubio L, Barguilla I, Domenech J, Marcos R, Hernández A (2020) Biological effects, including oxidative stress and genotoxic damage, of polystyrene nanoparticles in different human hematopoietic cell lines. J Hazard Mat 398:122900. 10.1016/j.jhazmat.2020.122900 32464564

[CR76] Sarasamma S, Audira G, Siregar P, Malhotra N, Lai YH, Liang ST, Chen JR, Chen KHC, Hsiao CD (2020). Nanoplastics cause neurobehavioral impairments, reproductive and oxidative damages, and biomarker responses in zebrafish: throwing up alarms of widespread health risk of exposure. Int J Mol Sci.

[CR77] Sgier L, Freimann R, Zupanic A, Kroll A (2016). Flow cytometry combined with viSNE for the analysis of microbial biofilms and detection of microplastics. Nat Commun.

[CR78] Shang L, Nienhaus K, Nienhaus UG (2014) Engineered nanoparticles interacting with cells: size matters. J Nanobiotechnol 12(5)1e11. 10.1186/1477-3155-12-5 PMC3922601 24491160

[CR79] Shi Y, Xu L, Tang J, Fang L, Ma S, Ma X, Nie J, Pi X, Qiu A, Zhuang S, Liu N (2017). Inhibition of HDAC6 protects against rhabdomyolysis-induced acute kidney injury. American Journal of Physiology-Renal Physiology.

[CR80] Sibarani J, Tjahjodjati T, Atik N, Rachmadi D, Mustafa A (2020). Urinary Cytochrome C and Caspase-3 as Novel Biomarker of Renal Function Impairment in Unilateral Ureteropelvic Junction Obstruction Model of Wistar Rats. Research and Reports in Urology.

[CR81] Stone V, Johnston H, Clift MJ (2007). Air pollution, ultrafine and nanoparticle toxicology: cellular and molecular interactions. IEEE Trans Nanobiosci.

[CR82] Sussarellu R, Suquet M, Thomas Y, Lambert C, Fabioux C, Pernet MEJ, Le Goïc N, Quillien V, Mingant C, Epelboin Y (2016) Oyster reproduction is affected by exposure to polystyrene microplastics. Proc Natl Acad Sci Unit States Am 113, 2430e2435. 10.1073/pnas.1519019113 PMC4780615 26831072

[CR83] Thompson RC, Swan SH, Moore, C.J.&Vom Saal, F.S.  (2009). Our plastic age. Philos. Trans R Soc Lond B Biol Sci.

[CR84] Walczak AP, Hendriksen PJ, Woutersen RA, Van der Zande M, Undas AK, Helsdingen R, Van den Berg HH, Rietjens IM, Bouwmeester H (2015). Bioavailability and biodistribution of differently charged polystyrene nanoparticles upon oral exposure in rats. J Nanopart Res.

[CR85] Walczak AP, Kramer E, Hendriksen PJ, Helsdingen R, van der Zande M, Rietjens IM, Bouwmeester H (2015). In vitro gastrointestinal digestion increases the translocation of polystyrene nanoparticles in an in vitro intestinal co-culture model. Nanotoxicology.

[CR86] Wang F, Bexiga MG, Anguissola S, Boya P, Simpson JC, Salvati A, Dawson KA (2013). Time resolved study of cell death mechanisms induced by amine-modified polystyrene nanoparticles. Nanoscale.

[CR87] Wang T, Wang L, Li X, Hu X, Han Y, Luo Y, Wang Z, Li Q, Aldalbahi A, Wang L, Song S, Fan C, Zhao Y, Wang M, Chen N (2017). ACS Appl Mater Interfaces.

[CR88] Warner TD, Mitchell JA (2002). Cyclooxygenase-3 (COX-3): filling in the gaps toward a COX continuum?. Proc Natl Acad Sci.

[CR89] Watts AJ, Urbina MA, Goodhead R, Moger J, Lewis C, Galloway TS (2016). Effect of microplastic on the gills of the shore crab Carcinusmaenas. Environ Sci Technol.

[CR90] Wright SL, Kelly FJ (2017). Plastic and human health: A micro issue?. Environ Sci Technol.

[CR91] Wünsch JR (2000) Polystyrene: Synthesis, Production and Applications. Rapra Technology, Shropshire 1e28.

[CR92] Wu X, Pan J, Li M, Li Y, Bartlam, Wang Y (2019) Selective enrichment of bacterial pathogens by microplastic biofilm. Water Res 165:114979. 10.1016/j.watres.2019.114979 31445309

[CR93] Yang Y, Yang J, Wu WM (2015). Biodegradation and mineralization of polystyrene by plastic-eating mealworms: Part 2. Role of gut microorganisms. Environ Sci Technol.

[CR94] Yin M, Jiang N, Guo L, Ni Z, Al-Brakati AY, Othman MS, Moneim AEA, Kassab RB (2019) Oleuropein suppresses oxidative, inflammatory, and apoptotic responses following glycerol-induced acute kidney injury in rats. Life sciences, 232, p.116634. 10.1016/j.lfs.2019.116634 31279782

[CR95] Yooeun C, Dokyung K, Shin WK, Youn-Joo A (2018) Trophic transfer and individual impact of Nano-sized polystyrene in a four-species freshwater food chain. Sci Rep 8, 1e11. 10.1038/s41598-017-18849-y PMC5762726 29321604

[CR96] Yousif E, Haddad R (2013). Photodegradation and photostabilization of polymers, especially polystyrene: Review. Springerplus.

[CR97] Zhao Y, Sun X, Zhang G, Trewyn BG, Slowing II, Lin VSY (2011) Interaction of mesoporous silica nanoparticles with human red blood cell membranes: size and surface effects. ACS Nano 5, 1366e1375. 10.1021/nn103077k 21294526

